# The modal basis of scientific modelling

**DOI:** 10.1007/s11229-023-04063-z

**Published:** 2023-02-16

**Authors:** Tuomas E. Tahko

**Affiliations:** Department of Philosophy, University of Bristol, Cotham House, Cotham Hill, BS6 6JL Bristol, UK

**Keywords:** Models, Modality, Counterfactuals, Truthmaking, Superheavy elements

## Abstract

The practice of scientific modelling often resorts to hypothetical, false, idealised, targetless, partial, generalised, and other types of modelling that appear to have at least partially non-actual targets. In this paper, I will argue that we can avoid a commitment to non-actual targets by sketching a framework where models are understood as having networks of possibilities as their targets. This raises a further question: what are the truthmakers for the modal claims that we can derive from models? I propose that we can find truthmakers for the modal claims derived from models in actuality, even in the case of supposedly non-actual targets. I then put this framework to use by examining a case study concerning the modelling of superheavy elements.

## Models, modality, and reality

The role of models and modelling in scientific research has been studied exhaustively by philosophers of science, and increasingly so in the last couple of decades. It is safe to say that a consensus exists that models can be used to *understand* and *explain* the world (e.g., Giere, 2004; Bokulich, 2011; Weisberg, 2013). But insofar as there is some type of connection between models and reality, we need to address what it is *in reality* that models represent. As Elliott-Graves (2020: 2) puts it: ‘in the simplest cases of model-world relations, target systems are *parts of the world*’. But do models always have actual targets or do they sometimes represent merely possible, non-actual phenomena? Moreover, if models supposedly do have non-actual targets, how do we assess the truth-conditions of claims that are made on the basis of these models? After a discussion of various ‘loose ends’, such as hypothetical models, Elliott-Graves (2020: 19) concludes that we may wish to adopt ‘a pluralistic attitude towards the ontological status of target systems’. This leads to difficult questions about the *modal* basis of scientific modelling, where the central issues concern the nature and justification of the modal content of claims made on the basis of models. More succinctly, we may divide these questions into the following two categories:^1^

1) The modal content that claims about actual targets have;

2) The modal content that claims about non-actual targets have.

Questions falling into both categories have received some attention in the philosophical literature, but often only in passing. The main hypothesis of this paper is that we can find actual truthmakers (i.e., truthmakers that exist in the actual world) for all true claims derived from models, both in the case of (1) and (2), i.e., actual and supposedly non-actual targets. This enables a unified treatment of a variety of seemingly different types of models, including those that sometimes appear to have non-actual targets, such as fictional, “false”, and highly idealised models. The upshot is that *all* models can be regarded as representing networks of possibilities and having actual targets, namely, the modal properties of actually existing entities. Accordingly, we can resist pluralism about the ontological status of target systems.

I should immediately add that this is to be understood as a metaphysical rather than an epistemic claim: I will not be concentrating on how we justify our beliefs in the modal claims derived from models, but rather on what, if anything, makes those claims true. Accordingly, the focus is on modal metaphysics and the associated framework of truthmaking rather than on modal epistemology. I do not mean suggest that modal epistemology is independent of the metaphysical framework though – in fact, I am sympathetic to the idea that modal metaphysics guides modal epistemology, as defended, in one guise, by Mallozzi (2021). The thought here is that before we can examine how modal claims derived from models are justified, we need to have some idea about what these modal claims are *about*. The proposed view is that they are about (networks of) possibilities that are grounded in the modal properties of actual entities. The content of these ‘modal properties’ will be specified in the course of the paper, but I will ultimately remain largely neutral on this issue; they could be, for instance, essential properties, dispositional properties, powers, or similar. However, this clearly suggests an anti-Humean approach to modality, even though I should hope that some aspects of the framework could be of use also to those with Humean sympathies.

Before I put my analysis forward, I will survey some of the existing literature and present a brief overview of some of the more general questions surrounding representation, idealisation, and fictionalisation.

Models are generally taken to represent their targets in virtue of being similar or isomorphic to them, but there are some complications (see, e.g., Suárez, 2003; Frigg and Nguyen, 2021). I will assume that scientific models represent but will remain largely neutral about the representation relation itself, while accepting that representation will likely often involve at least partial similarity or isomorphism. Furthermore, the representational approach to models doesn’t necessarily need to entail a representational relationship between a model and an actual, real world target system (for discussion see Knuuttila and Boon, 2011). As already noted, scientific modelling is sometimes considered to involve non-actual targets and it’s not clear whether the usual representational approach to modelling can capture these cases (cf. Grüne-Yanoff, 2013). For instance, it is unclear whether *hypothetical models* can be considered to have real world targets. As Weisberg puts it in his important book on modelling:

*Ex hypothesi*, their targets are nothing at all. With a little more nuance, we can say that the targets of hypothetical models are possibilities. Explaining how hypothetical models can tell us about such possibilities would require a lengthy discussion about the metaphysics of possibilities, which is beyond the scope of this book. (Weisberg, 2013: 121.)

Indeed, we do need a discussion about the metaphysics of possibilities. Specifically, we need a discussion of what the relevant possibilities are and how we can assess the counterfactual propositions that can be derived from these models/possibilities. As already indicated, I think that the best way to understand models is that they *represent* possibilities rather than *are* possibilities, whatever the latter may mean. So, in this paper, I will work with the assumption that models quite generally – not just hypothetical models – represent possibilities.^2^ I leave the nature of the relevant modal space largely open, but personally I favour a realist framework, which entails a commitment to a form of objective modality (rather than, say, epistemic modality). Ultimately, the relevant space of possibility that scientific models are concerned with needs to be restricted: mere logical possibility is too unrestricted, whereas physical/nomological possibility may sometimes be too restrictive. For instance, highly idealised models may involve physically impossible scenarios, and sometimes we may not be fully aware about the relevant nomological constraints, e.g., when modelling string theory or other highly speculative theories concerning fundamental physics.^3^ On the other hand, some modelling activities may require much more tightly restricted modal spaces. Accordingly, the correct restriction also depends on the model’s target. I will primarily be discussing cases from physics, which often involve a rather broad modal scope.

To summarise: the upshot of the paper is that all models represent networks of possibilities, and these possibilities are grounded in the modal properties of actual entities. Accordingly, we can avoid a commitment to non-actual targets. But what are the alternatives and why is this approach worth pursuing? In brief, if we postulate, e.g., Lewisian possible worlds, some kind of abstract entities (e.g., fictions), or perhaps even impossible worlds to account for the relevant modal claims then it seems that the target systems cannot be actual. Or if they are, we need some explanation of how, exactly, the relevant modal metaphysics is connected to actuality. On the view that I favour, where the modal claims are made true by the modal properties of actual entities, we can avoid non-actual targets and present a unified account of all modal claims derived from models. Now, this does admittedly require a further analysis of the relevant modal metaphysics. As already indicated, I favour an anti-Humean approach, which could for instance be essentialist, dispositionalist or powers-based (cf. Borghini and Williams, 2008; Lowe, 2008; Jacobs, 2010; Tahko Forthcoming). However, I would like to remain as neutral as possible here, so I will not be defending any of these options in more detail.

I will begin, in Sect. 2, by briefly outlining challenges posed by “false” models and fictional models, which are sometimes considered to have non-actual targets. Section 3 puts forward the main claim of the paper, which is that we can generally find the relevant truthmakers for modal claims based on modelling in actuality, regardless of the type of model in question. In Sect. 4, I will present a case study concerning the modelling of superheavy elements, before concluding in Sect. 5.

## “False” models and fictional models

The first complication concerning the relationship between models and reality is that we sometimes see reference to “false” models, which are at least sometimes capable of explaining or providing understanding (cf. Wimsatt, 1987; Bokulich, 2011; Mäki, 2012). Strictly speaking, we should perhaps not talk about the “truth” or “falsity” of models themselves.^4^ On the view to be developed in this paper, whereby models represent possibilities, we can say that models at least sometimes seem to represent possibilities that do not correspond with actual world targets, i.e., they represent non-actual possibilities. In that way, we could say a model is “false”, as it does not represent an *actualised* possibility. But this does not really mean that our model “falsely” represents, since it may have been our aim all along to construct a model of a *non-actual* possibility. So, we might say of the same model that it is “true” because it accurately represents that non-actual possibility. But does this entail that the model has a non-actual target? Not automatically: we must ask a further question about the basis of the relevant possibility. Accordingly, we need to slightly reinterpret the usual language of true/false in this context, given that truth is generally taken to be factive and hence to entail actuality, but a model may truly and accurately represent a non-actual possibility as well. Nevertheless, such a non-actual possibility may be grounded in properties that actual entities have, as I will go on to suggest.

The underlying question here is how can models that represent non-actual possibilities provide explanations that relate to *actual* phenomena? It may be useful to specify here that, when considered as a network of possibilities, even a highly idealised model is likely to represent *some* actual phenomena (but there may be exceptions). Let me illustrate this by discussing Bokulich’s account:^5^

[I]n order for a model M to explain a given phenomenon P, we require that the counterfactual structure of M be isomorphic in the relevant respects to the counterfactual structure of P. That is, the elements of the model can, in a very loose sense, be said to “reproduce” the relevant features of the explanandum phenomenon. (Bokulich, 2011: 39.)

A key part of Bokulich’s account is the reliance on isomorphism of *counterfactual structure* – this is why models are also able to give us information about how a target system would behave if we were to change some elements of the system. Since I treat models as representing possibilities, we may understand the counterfactual structure itself to “be” the possibility (or more accurately, a network or possibilities) being modelled. Bokulich doesn’t specify the nature of the counterfactual structure in much detail, but I suggest that we should understand it in terms of dependencies between the entities – such as substances and properties – involved in the structure.^6^ In other words, where a given model represents a network of possibilities via its counterfactual structure M, this structure can be said to be isomorphic in the relevant respects to an actual dependence structure P, where this dependence structure is given by the dependence relations that obtain between the entities involved in P.

Admittedly, at this abstract level, it is difficult to grasp how we are supposed to put this idea to use, so let me give some more detail regarding Bokulich’s account to make this clearer. Bokulich’s own focus is on the explanatory function of models and it’s clear that this explanatory function needs to be undergirded by something like what she proposes in the quoted passage, i.e., by some connection, such as isomorphism, between the elements of the model and an actual target phenomenon. There are models that do not have this type of explanatory aim but instead only a pragmatic or instrumental function in scientific practice. Such models are not my focus here. As Bokulich specifies: ‘An *explanatory model*, by contrast [to models with an instrumental function], does aim to give genuine insight into the way the world is’ (2011: 44).

However, since explanatory models may also be used to describe *non-actual* phenomena, such as systems that are physically impossible given the known laws of physics, there is a further question about how such models could be connected to actual target systems. In some cases, the answer seems relatively straightforward. For instance, the ideal gas model or a model involving a frictionless plane are best understood as *idealisations* of actual phenomena; they generally involve many of the same laws that govern actual phenomena. Even though we know, given the actual laws of physics, that the behaviour these models describe could not take place exactly as described, there is arguably a clear isomorphism between such idealised models and real world systems: the relevant *entities* involved and at least most of the dependencies between them are exactly the same in the idealised model, but we ignore or change some of the actual laws that apply to them. Accordingly, the relevant possibilities that such idealised models represent may still be grounded in the modal properties of actual entities. This may be considered to be a type of *de-idealisation*.

But what if there is no easy route back to actual entities? *Fictional models* may seem to resist the type of de-idealisation that we have just discussed, so it may be more difficult to see how such models could have actual targets. The role of fictions in modelling has received plenty of attention (already in Cartwright, 1983; and also, e.g., Frigg, 2010; Bokulich, 2011; Tee, 2019; Kimpton-Nye, 2020). Let me continue with Bokulich, who outlines an example of an explanatory fictional model: Bohr’s atom. I shall assume that Bokulich is right about the reasons that she states for classifying Bohr’s model of the hydrogen atom as a *fiction* – she bases this on the fact that the electron orbits described by the model are ‘classical’ while we know from modern quantum mechanics that this is certainly incorrect; electron orbitals do not follow definite classical trajectories and are instead better described as clouds of probability density around the nucleus (Bokulich, 2011: 42). Importantly, Bokulich argues that the trajectories in Bohr’s model cannot be understood as idealisations because there is no way to de-idealise them, i.e., ‘one cannot simply add something back in to the Bohr orbits that was left out or change some parameter of the orbit to recover the correct quantum description’ (ibid., 43). Nevertheless, there is still a way to extract information about the behaviour of actual hydrogen atoms from Bohr’s model, because ‘the counterfactual structure of Bohr’s model is isomorphic to the counterfactual structure of the spectral phenomena’ (ibid.). Again, I take it that Bokulich is right about all this.

The upshot would then be that even in the case of fictional models as Bokulich understands them there seems to be a way to preserve the connection between models and reality, where ‘reality’ is understood as the counterfactual structure of actual phenomena, i.e., the manner in which a system would behave if something were to be changed. Accordingly, the key question is how we should interpret the relevant possibility, i.e., “if something were to be changed” regarding the actual phenomena. If this possibility can be grounded in the modal properties of actual entities, then there would seem to be no need to postulate a non-actual target. In the next section, I will sketch a framework for interpreting the relevant modal claim so that we have the tools to analyse models that involve similar modal claims.

## Truthmakers for modal modelling

According to the framework being developed in this paper, the question about what makes modal claims derived from scientific models true is a special case of modal metaphysics. Specifically, since we have seen in the previous sections that models often seemingly fail to have direct representational links to real world phenomena – and instead provide explanations by representing possibilities that replicate some aspects of the actual dependence structure – we should employ the toolbox of modal metaphysics and epistemology to understand the relevant truthmakers of the propositions derived from such models.^7^

We need to complement this toolbox with one further distinction before we are in a position to formulate the core questions that the present framework aims to address, namely, *model-system claims* and *target-system claims*. Model-system claims concern the representations in models, e.g., a frictionless plain representation. Target-system claims concern the phenomena being represented. In the case of a frictionless plain there is nothing in the actual world that corresponds to it, given that no frictionless plains exist. So there cannot be a representation *of* an actual frictionless plain, even if there is a frictionless plain representation in a model; this is why we might need to consider non-actual target systems. Accordingly, we can ask two types of questions about the truthmakers of such claims. My proposal is that, in both cases, we can find these truthmakers in the actual world, where truthmakers are entities such as objects, properties and relations.^8^ Following this outline, we may divide the core questions regarding the role of truthmaking as follows:^9^

(i) What are the truthmakers of model-system claims?
(ii) What are the truthmakers of target-system claims?
(iii) Are the truthmakers of non-actual target-system claims the same as the truthmakers of actual target-system claims?
(iv) Does the ontology of model systems make a difference to the truthmakers of target-system claims?

It should now be clear that my answer to (i) and (ii) is the same: the truthmakers of both model-system claims and target-system claims are actual entities. My answer to (iii) is affirmative. I favour a unified ontology of target-systems, namely, the target-systems are networks of possibilities. The possibilities are grounded in the modal properties of actual entities, which also entails a unified treatment of the relevant target-system claims. My answer to (iv) is negative: all model systems should receive the same, unified treatment, i.e., they should all be considered to represent networks of possibilities, which takes us back to the previous questions.

But how does this picture fare with some actual examples of modelling? Consider examples like economic or climate modelling, both of which notoriously deal with “falsities”, or even *impossibilities*, e.g., claims that involve idealisation that clearly conflicts with the known laws of nature: infinite velocities, perfect information, zero transaction costs, etc. (Mäki, 2012).^10^ In fact, it is common to think that models almost always – or ‘almost by definition’, as Mäki (2012: 216) puts it – involve “false” elements in this sense. I have already discussed some of the various strategies to reconcile this fact with the representational approach to models, and the sense in which the true/false distinction does not neatly fit the present account, but it’s worth taking a moment to analyse how we should understand these cases in terms of the proposed framework.

The starting point of the analysis is on a *delimitation* on what we aim to model, i.e., a delimitation of the network of possibilities. For this purpose, we need a space of objective possibility that is narrower than that of conceptual or logical possibility. Why? Because otherwise there will be no actual truthmakers for the target-system claims. To see this, consider a metaphysically impossible claim, like ‘gold could turn out not to be an element’. When this is understood as a metaphysical, target-system claim, it of course has no actual truthmaker. But since I have argued that target-system claims should also have actual truthmakers, we need some method of either ruling out such metaphysically impossible claims or finding an alternative way to secure truthmakers for them. Accordingly, I suggest that we should restrict the metaphysical claims we make by considering the relevant metaphysically necessary essentialist truths, such as the truth about gold being an element. Now, the tricky thing is of course to establish the relevant delimitation, that is, the space of metaphysical possibility. But assuming that this can be done, we also ought to restrict our modelling activities in terms of this type of metaphysical delimitation, taking the first step toward a more adequate restriction of possibility judgments arising from scientific models.^11^

The case of gold serves as toy example, but to put this idea to use, let me return to an example mentioned above, from Bokulich: Bohr’s atom. Bokulich’s analysis treats this model as an explanatory fictional model, partly because there is no plausible de-idealisation available that would enable us to recover the modern quantum-mechanical description of the hydrogen atom from the Bohr model. In the context of the present proposal, a somewhat different strategy is needed, as the goal is to identify the actual truthmakers of the target- and model-system claims. However, Bokulich’s analysis of the Bohr model is nevertheless quite helpful for my purposes, for she has already identified a number of truthmakers for the claims that we can make on the basis of the model (which she calls ‘what-if-things-had-been-different questions’). These involve questions like how the emission spectrum of (actual) hydrogen would change if the electron orbits were elliptical rather than circular, or how the spectral lines would change if the hydrogen atom were placed in an external electric field (Bokulich, 2011: 43). In other words, we can make true target-system claims about the behaviour of actual hydrogen atoms on the basis of the Bohr model. This is of course a clear indication of the model’s usefulness, but it also gives us a fairly straightforward account of the truthmakers of the relevant counterfactuals that we can state based on the model: the actual properties of hydrogen (and its composites) are what make these claims true.

On the face of it, this may appear to be in tension with Bokulich’s own treatment of the case, since she makes it clear that there is no straightforward de-idealisation of the Bohr model. However, I would suggest that we can still find a route to relevant truthmakers in the actual world. This is because the relevant counterfactual claims, like the one about electron orbits or spectral lines, do hold fixed a number of the actual properties of hydrogen as well as the laws that apply to them, such as hydrogen’s nuclear charge and the mass and charge of the electrons. Moreover, since these actual properties are held fixed in the model, they also help restrict the relevant space of possibility. Admittedly, there are some aspects of the model that cannot be so easily handled, but this is to be expected given that some aspects of the model violate the actual laws of nature.

Accordingly, it’s important to see here that only a subset of the modal claims made on the basis of the Bohr model, like with any model, may be analysed in this way – this is another source for delimitation. For instance, given the uncertainty principle, we are not going to find truthmakers for any propositions involving known electron orbits and locations, despite these being derivable from (or assumed in) the Bohr model. The upshot is that the same model can enable both true and false counterfactual claims. This is of course as it should be because models are often designed precisely for this purpose, i.e., to correctly answer a range of ‘what-if-things-had-been-different questions’ while being unable to do so for others. The trouble is that sometimes we do not know which questions are correctly answerable and which ones aren’t.

Let me conclude this section by considering some borderline cases. I mentioned above that we might want to rule out models that represent (supposedly) metaphysically impossible scenarios, such as a model that leads us to infer that gold is not an element. There are somewhat more striking examples as well, such as the case of *counterlegal models*. Counterlegals are counterfactuals with physically impossible antecedents, such as ‘if faster than speed of light travel were possible, Einstein’s special relativity would be false’. If we assume that superluminal travel is indeed impossible (like special relativity is thought to dictate), then this is a genuine counterlegal. Why should we be interested in such cases? In metaphysical contexts, counterlegals feature quite frequently: we often consider what the world would look like if the laws of nature or the values of fundamental constants that feature in them were slightly tweaked (e.g., Lange, 2008; Tahko, 2015a). But counterlegals do also feature in scientific practice, and arguably such counterlegals are not just *vacuously* true (as examined by Tan, 2019; Kimpton-Nye, 2020; McLoone, Grützner & Stuart, 2023). For instance, Tan (2019: 40) mentions examples like:

(D) If diamond had not been covalently bonded, then it would have been a better electrical conductor (Tan, 2019: 40).

Tan in fact regards the antecedent in (D) to be *metaphysically* impossible and not just physically impossible – this is plausible enough if the microstructure of diamond is regarded as essential to it (for discussion regarding the plausibility of such microstructural essentialist picture, see Tahko 2015b). I will set aside the additional issue regarding the vacuousness of counterlegals and counterpossibles, i.e., the issue of whether counterfactuals with (either physically or metaphysically) impossible antecedents are vacuously true.^12^ For what it’s worth, my sympathies are with those who side with non-vacuousness, such as Tan, and I shall assume that he is right in stating that:

‘the success of numerous scientific endeavors—scientific explanation, model-based reasoning, and reasoning about superseded theories—requires using counterfactuals which frequently turn out to have metaphysically impossible antecedents’ (Tan, 2019: 38).

If this is correct, then we obviously need to address counterlegal modelling in our overall theory of scientific modelling. One specific problem that metaphysically impossible antecedents create – and the one that is of most interest to the general framework being developed in this paper – concerns the truthmakers of the relevant counterlegals. My proposal is that we can deal with this problem in the same way as we deal with other non-actual possibilities, i.e., the truthmakers for these counterlegal claims, insofar as they have any, can be found in the actual world.

How should we analyse cases like (D)? I propose that we should start with what we know about actual cases of electrical conductors. Covalently bonded molecules are poor electrical conductors, whereas, say, compounds involving metallic bonds have a high electrical conductivity. So, there are in fact many plausible truthmakers for (D): any actual non-covalently bonded compound that conducts electricity will presumably do. This isn’t changed by the fact, if it is a fact, that it is metaphysically necessary that diamonds are covalently bonded. I would instead propose that the most natural way to read a claim like (D) does not have anything to do with diamonds, but it is rather about the phenomenon of electrical conductivity. Accordingly, my proposal is to approach metaphysically impossible models, or models that involve counterpossibles, on a case by case basis. Some of them may be deemed as failing to model anything. But in many cases, such as, perhaps, with (D), there might be a plausible analysis that allows us to identify actual truthmakers.

## Modelling superheavy elements

Now that we’ve discussed a number of examples in passing, it might be useful to take a closer look at one interesting case. This example is inspired by Lowe’s brief discussion concerning transuranic elements – elements with an atomic number higher than 92, i.e., that of uranium:

Scientists trying to discover the transuranic elements knew before they found them what it was that they were trying to find, but only because they knew that what they were trying to find were elements whose atomic nuclei were composed of protons and neutrons in certain hitherto undiscovered combinations. (Lowe, 2008: 41.)

The important point regarding this example is that in many cases, the existence of a transuranic element was predicted – and its properties very accurately modelled – before it was actually synthesised (see Kragh, 2013 for an overview). This is especially true for *superheavy* elements, those with an atomic number greater than 103, which have never been observed in nature, outside the laboratory (not for want of trying). Even when these elements are created and observed in the laboratory, some of them have a very fleeting existence, measured in microseconds. One of the most important advances toward modelling superheavy elements came from the mathematician Maria Goeppert Mayer, whose model connected the orbits of neutrons and protons on their shells with the particles’ spins (Chapman, 2020: 5). It soon emerged that some combinations of protons and neutrons appear to produce higher stability of the nucleus (these are combinations of protons or neutrons arranged into complete shells within the atomic nucleus). Eugene Wigner coined the term ‘magic numbers’ for this discovery; the currently recognised ‘magic numbers’ are 2, 8, 20, 28, 50, 82, and 126 – the last of these corresponding to the hypothetical element 126, *unbihexium*. Currently, the heaviest element synthesised is Z = 118, which has been named *oganesson*.^13^

**Fig. 1:**
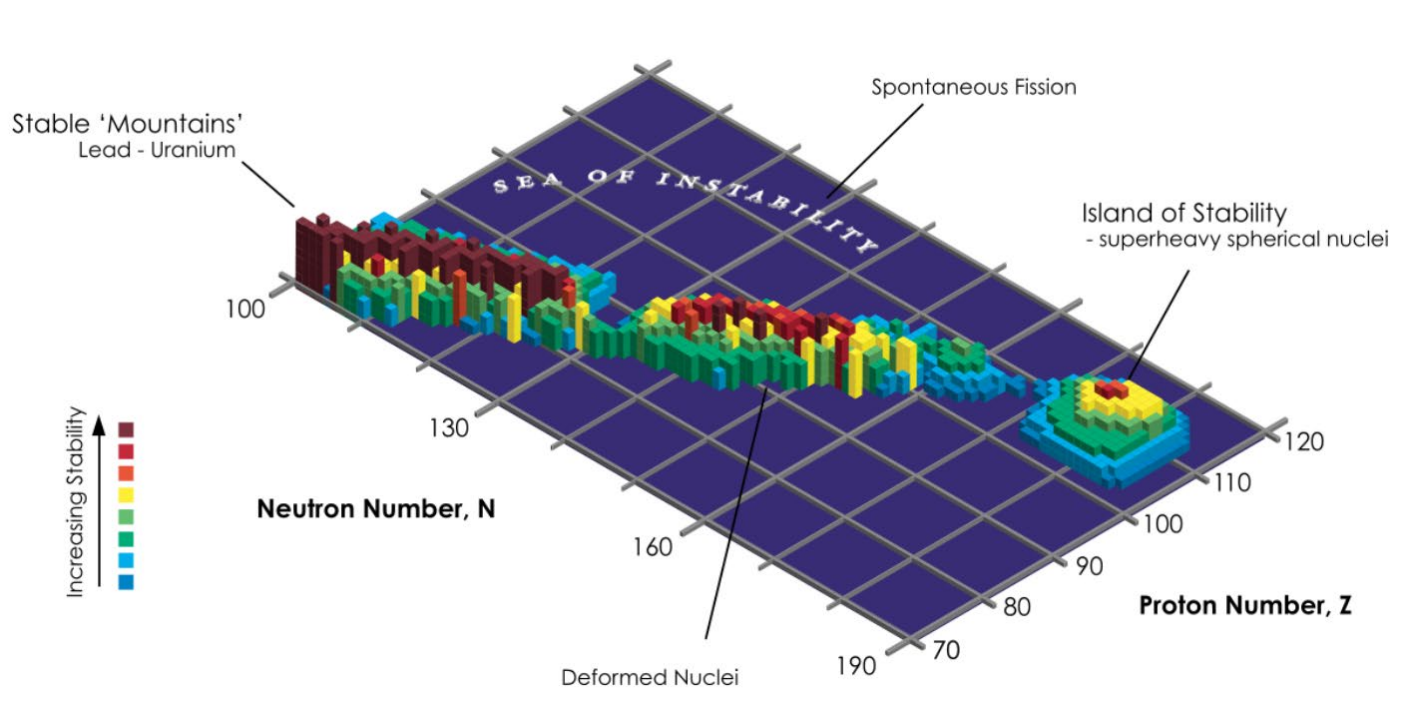
Island of Stability. Wikimedia Commons CC BY-SA 3.0 (unmodified). Available from https://commons.wikimedia.org/wiki/File:Island-of-Stability.png

The history of the search for superheavy elements is full of interesting details (see Kragh, 2013; Chapman 2020), but I’d like to focus on one particular aspect now, namely, the fabled ‘island of stability’, pictured in Fig. 1. The existence of such an island has been predicted on the basis of the ‘magic numbers’, where Z = 126 is of particular interest. The reason for this is that the expected longer half-life of Z = 126 could even result in some practical applications (compared to the extremely short half-life of the most long-lived isotope of *oganesson*, oganesson-294, which has a half-life of about 0.89 milliseconds).

Without going into too much technical detail, we can easily extract some promising counterfactuals from the efforts to model the island of stability. Here’s one:

(U) If *unbihexium* existed, it would have a longer half-life than *oganesson*.

(U) is presumably true, at least if *unbihexium* is ‘doubly magic’, i.e., has an isotope with a neutron number equal to one of the magic numbers, in addition to the proton (atomic) number. But any model that can be used to derive target-system claims such as (U) may seem to involve at least a partially *non-actual target*, since no atoms of unbihexium exist in the actual world. So, the question that we are now faced with is how could there be an actual truthmaker for (U), like the present framework requires, given that no samples of *unbihexium* exist and indeed it could be that limitations in technology might prevent us from ever synthesising any?

The answer is magic! More precisely, the suggestion is that (U) is true because the unexpectedly strong stability familiar to us from other cases involving magic numbers points toward a systematic pattern. One of the clearest examples of this is the case of calcium (Z = 20), which has two ‘magical’ isotopes, with neutron numbers 20 and 28. Compared to the binding energy calculated from the Weizsaecker formula, which is commonly used to calculate the binding energy of nuclei, both of these isotopes have a much higher binding energy than expected. Of course, ultimately, we need to explain *why* it is that isotopes involving these magic numbers have higher than expected stability. The answer to this question points toward the shell model of the nucleus.

**Fig. 2:**
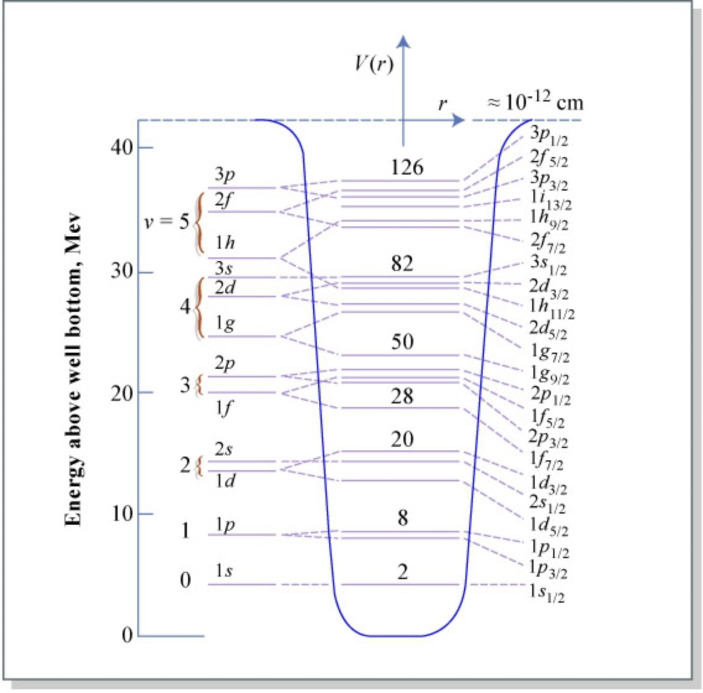
Energy Levels of Nucleons in a Smoothly-Varying Potential Well. Figure by MIT OpenCourseWare. From Meyerhof. CC BY-NC-SA 2.0 (unmodified). Available from https://www.flickr.com/photos/mitopencourseware/3772864128

The shell model of the nucleus is rather more difficult to visualise than the more familiar electron shell model. A key issue here is how it can be possible for the very densely packed nucleons to complete their orbits without colliding. However, we should not of course try to conceive of this classically, just like we saw in the case of Bokulich’s commentary of Bohr’s model. Fortunately, a lot of effort has gone into modelling all this. Figure 2 shows the energy levels of nucleons in a potential well and connects this to the ‘magic numbers’ of nucleons, where these indicate closed shells. So, as a matter of fact, here we have a very detailed account of what could make (U) true. There is also a basis here for many other counterfactuals that we might extract from the shell model of the nucleus. It is of course the shell model which also underlies the efforts to model the island of stability. The truthmakers for (U) and the like are thus the actual energy states of the nucleons and their positions in the potential well, which gives rise to higher stability in cases where we have closed shells, as indicated by the ‘magic numbers’ of nucleons. Accordingly, the relevant counterfactual structure can be extracted from the dependencies modelled in the shell model of the nucleus, which is also, and especially, able to tell us about cases like (U), given that unbihexium (Z = 126) corresponds to one of the ‘magic numbers’.

Let me summarise the resulting picture. There are actual dependencies among entities in the world which are the truthmakers for claims like (U). These concern, for instance, the ‘magic numbers’, which we are familiar with from other actual entities. So, the relevant entities here are neutrons and protons and their relative numbers, which vary according to a known structure of binding energies and energy levels, giving rise to certain dependencies (partially pictured in Fig. 2). There is of course plenty more scientific detail to all this, but we can instead focus on the core philosophical idea, whereby the actual entities that can be identified as the relevant truthmakers may be divided into the following two categories:

(1) the relevant entities, i.e., substances (if one accepts substances in their ontology) and properties, which in this case are the nucleons and their energy levels;

(2) the dependence structures that these entities participate in, which in this case involves the shell model of the nucleus, i.e., where the shell that a nucleon enters into depends on how many nucleons there are altogether, and this influences the energy levels and ultimately the stability of the nucleus.

While this picture is vastly simplified, the relevant details to be filled in are primarily empirical. The point is that with the information that we have about actual nuclei and the manner in which their stability is determined (via the shell model), we can apply an already familiar pattern to cases like unbihexium. Accordingly, the relevant truthmakers for claims like (U) can indeed be found in actuality (if they are true at all).

## Conclusion

One initial hypothesis of this paper was that all true claims based on models have actual truthmakers. I have presented a defence of this hypothesis with an appeal to a framework whereby each scenario derivable from a model may be understood as a network of possibilities, arising from the dependence structures among the participating entities. I then suggested that the truthmakers of both the target-system and the model-system claims that we can make can be found in the actual world, namely, the objects, properties and relations that actually exist. Finally, I have put this framework to use in an analysis of the intriguing modelling efforts concerning superheavy elements and the island of stability. Much remains to be discussed of course. In particular, I have not put forward a detailed picture about how we in fact gain knowledge about the truthmakers for the modal claims derived from models. But one thing is clear: on the picture at hand, the epistemology of modal modelling will become a (special) case of the epistemology of modality.

In this regard, I’d like to briefly point out a connection to the existing literature on modal epistemology, namely, Bob Fischer’s (2016, 2017) theory-based approach.^14^ Fischer suggests that since we don’t have any special faculty of modal intuition, ‘our best shot is to make inferences from actuality to possibility, to offer analogical and inductive arguments, and to do our best to figure out the world’s rules’ (Fischer, 2016: 231). Perhaps the most relevant part of Fischer’s approach for the present application is the idea that when one believes a theory to be true, one believes that ‘a class of models represents a system’ (2016: 237). On this line of thought, the reasons to believe, say, that unbihexium has a certain level of stability could be derived from the general belief that our theory regarding the structure of the periodic table and the magic numbers is true. The resulting picture of the epistemology of modal modelling that we could derive from this can thus quite easily be adapted to a case study such as the one regarding superheavy elements. However, it is worth noting that it has not been my attempt to address the general epistemology of modal modelling here; and conversely, Fischer’s theory-based epistemology of modality is not an attempt to analyse the truthmakers of our modal beliefs, but rather to explain *why* our modal beliefs are justified. What I share with Fischer is the attitude that we should put forward a *realist* epistemology. But such an epistemology also requires a realist *ontology* about what makes those justified modals belief *true*, and I have defended the thought that we might find the truthmakers in the actual world.^15^

## Footnotes

1 Thanks to an anonymous reviewer for suggesting this way of putting the distinction.

2 This of course already covers those models that represent actual systems, because actual systems are also possible. Thanks to an anonymous reviewer for highlighting this.

3 An anonymous reviewer makes the valid point that, in some cases at least, we could understand such highly speculative models as an attempt to set the stage in terms of logical possibility, as a first step (cf. also Baron and Colyvan, 2021, who examine explanations that appeal to logical impossibilities). I agree that we must start from somewhere, but I can’t easily think of serious models which would appeal *only* to logical possibility. Perhaps it would be better to say that logical consistency (assuming we know which logic to use, of course), is the first restriction.

4 Thanks to an anonymous reviewer for making this very helpful point, namely, that models themselves are not truthbearers, even though the inferences that we make about their targets may be true or false. I think this is absolutely righty, as I hope to make clearer in the course of this paper.

5 Even though Bokulich does not treat models as representing possibilities, her account can easily be adapted for my purposes, as I will make clear in what follows.

6 I will continue to talk in terms of entities like substances and properties, but another alternative would be to adopt a structuralist framework, such as the ontic structural realism defended by Ladyman and Ross et al.( 2007).

7 This type of *indirect* view of representation is of course a fairly standard approach to models, although there are those who favour a direct view (e.g., Toon, 2012; Levy 2015). See Frigg and Nguyen (2021) for further discussion.

8 Again, I continue to talk in terms of such entities, but if one doesn’t accept objects or substances, for instance, then this could be adapted. I should also note that some would regard only ‘facts’ to be capable of acting as truthmakers. This does not need to make much of a difference for the current proposal though, since on a plausible understanding of facts as complex ‘worldly’ entities, we can consider them to be individuated and ‘constituted’ by their worldly constituents, such as objects, properties, and relations. So, ultimately, these are the entities that enable truthmaking.

9 This list is partially reconstructed from the comments of an anonymous reviewer on an earlier draft of the paper, and I am grateful to them for helping me to greatly clarify the aims of the paper.

10 On economical modelling(see Mäki, 2012)where the apparent “falsity” of economic models is discussed; on climate modelling, see, e.g., the detailed account in Winsberg( 2018).

11 A related point has been made by Verreault-Julien (2019: 31), who notes that we have good reasons to think that scientists do not always ‘adequately restrict their possibility judgments’ when engaging in modelling activities, and indeed sometimes these possibility judgments are incorrect.

12 This issue is discussed in detail by Tan and also Kimpton-Nye (2020).For further discussion, see also Brogaard and Salerno (2013).

13 There is a nice continuity here with Bohr’s model of the atom, discussed earlier, but Bohr’s model left the nature of the nucleus open. Interestingly, Bohr predicted the electron structure of *oganesson* almost 100 years ago, although the existence of even normal transuranic elements was hardly even dreamt of at that point.

14 Thanks to an anonymous reviewer for pointing out this connection.

15 See Tahko Forthcoming for further discussion, including the relevant modal epistemology.
